# Circulating Tumor-Cell-Associated White Blood Cell Clusters in Peripheral Blood Indicate Poor Prognosis in Patients With Hepatocellular Carcinoma

**DOI:** 10.3389/fonc.2020.01758

**Published:** 2020-11-02

**Authors:** Qiong Luo, Chunming Wang, Bangjian Peng, Xiaoyu Pu, Lei Cai, Hangyu Liao, Kunling Chen, Cheng Zhang, Yuan Cheng, Mingxin Pan

**Affiliations:** ^1^Second Department of Hepatobiliary Surgery, Zhujiang Hospital, Southern Medical University, Guangzhou, China; ^2^Department of General Surgery, Affiliated Hengyang Hospital, Southern Medical University (Hengyang Central Hospital), Hengyang, China; ^3^Department of Hepatobiliary Surgery, The Fifth Affiliated Hospital of Southern Medical University, Guangzhou, China; ^4^SurExam Bio-Tech, Guangzhou Technology Innovation Base, Guangzhou, China

**Keywords:** CTC-WBC cluster, hepatocellular carcinoma, prognosis, Kaplan-Meier plot, circulating tumor cells

## Abstract

**Aim:** Circulating tumor cells (CTC) are a precursor to metastasis in several types of cancer and are occasionally found in the bloodstream in association with immune cells, such as white blood cells (WBCs). CTC-associated WBC (CTC-WBC) clusters can promote CTC appreciation and metastasis, suggesting that patients with CTC-WBC clusters found in the peripheral blood may have a worse prognosis. However, it is unclear whether CTC-WBC clusters are present in the peripheral blood of patients with hepatocellular carcinoma (HCC) and suggest a poor prognosis for HCC.

**Methods:** We collected peripheral blood from 214 patients with HCC from January 2014 to December 2016. CanPatrol™ CTC analysis technology was used to isolate and count CTCs and CTC-WBC clusters in the patients' peripheral blood. Chi-squared analysis was used to calculate the correlation between the CTC-WBC clusters and clinicopathological characteristics. Kaplan–Meier survival analysis and Cox regression analysis were used to assess patient prognosis.

**Results:** We used CanPatrol™ CTC analysis technology to count different types of CTCs and CTC-WBC clusters. The results showed that CTC-WBC clusters and tumor size (*P* = 0.001), tumor number (*P* = 0.005), portal vein tumor thrombus (*P* = 0.026), BCLC stage (*P* < 0.001), AFP level (*P* = 0.002), and total number of CTCs (*P* < 0.001) were statistically related. Cox regression analysis revealed that CTC-WBC clusters are an independent prognostic indicator of DFS (HR = 1.951, 95%CI:1.348–2.824, *P* < 0.001) and OS (HR = 3.026, 95%CI:1.906–4.802, *P* < 0.001) in HCC patients. Using Kaplan–Meier analysis, we found that positive CTC-WBC cluster patients had significantly shorter DFS and OS than patients with negative CTC-WBC (*P* < 0.001 and *P* < 0.001, respectively).

**Conclusions:** CTC-WBC clusters in the peripheral blood are an independent predictor of DFS and OS, and their presence indicates poor prognosis in patients with HCC.

## Introduction

Liver cancer is predicted to be the sixth most commonly diagnosed cancer and fourth leading cause of cancer-related death worldwide in 2018, with ~841,000 new cases and 782,000 deaths annually (1). China alone accounts for about 50% of the total number of cases and deaths (2). In the past two decades, despite various advances in the treatment of hepatocellular carcinoma (HCC), such as molecular-targeted therapy, radiofrequency ablation, and interventional embolization, surgery remains the most important treatment (3, 4). The high metastasis and recurrence rate of HCC indicates that its overall prognosis is still unsatisfactory (5–7). Metastasis and recurrence are the leading causes of death in patients diagnosed with invasive cancer. Tumor cells that leave the site of the primary tumor and are transported by circulation to distant organs are called circulating tumor cells (CTCs). CTCs are considered the source of tumor metastasis and recurrence (8–10).

The “seed and soil” hypothesis states that tumor cells (seeds) and stromal cells (soil) are involved in metastasis and clump together to form tumor microemboli (11, 12). It is a widely held view that the presence of tumor microemboli in the circulation is associated with a poor prognosis (13, 14). CTCs are precursors to metastasis in several types of cancer, occasionally appearing in clusters in the blood or found associated with immune-related cells, such as white blood cells (WBCs) (15). In some cases, the fact that CTCs clusters are “the soil with seeds” may help further transfer them to distant organs and continue to grow. However, it is unclear whether there are circulating tumor cell-associated white blood cell (CTC-WBC) clusters in the peripheral blood of patients with HCC and whether the presence of such CTC-WBC clusters is related to the prognosis of HCC.

In this study, we evaluated CTCs and CTC-WBC clusters in the peripheral blood of 214 preoperative HCC patients, starting in 2014. First, we used CanPatrol™ CTC analysis technology to label CTCs and WBCs with different markers and then counted CTCs and CTC-WBC clusters using fluorescence microscopy. The main purpose of this retrospective study was to determine the influence of CTC-WBC clusters on the risk of recurrence and metastasis, and thus determine whether CTC-WBC clusters are potential biomarkers for tumor recurrence and metastasis.

## Materials and Methods

### Study Design

**Figure 1:**
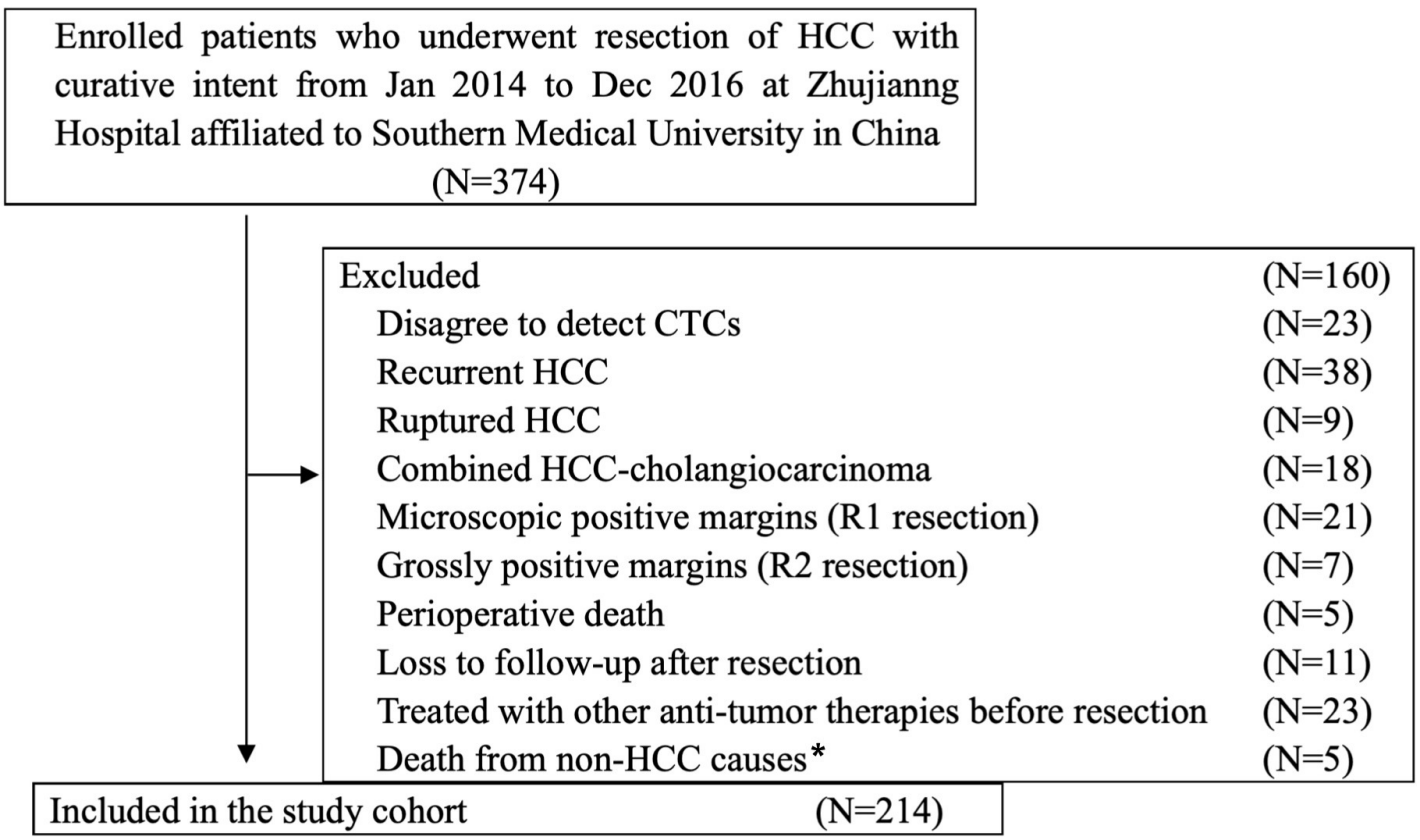
Flowchart of the diagnosis of patients enrolled in the study. *Death from non-HCC causes included 2 cases of cerebral hemorrhage, 2 cases of myocardial infarction, and 1 case of traffic accident.

From January 2014 to December 2016, 374 patients with HCC who underwent radical resection at the Zhujiang Hospital of Southern Medical University participated in this retrospective cohort study. The final 214 patients were screened according to inclusion criteria. The inclusion criteria were as follows (Figure 1): (a) HCC was diagnosed according to the World Health Organization's pathological standard (16); (b) patients did not have a relapsed or ruptured HCC, or cholangiocarcinoma; (c) patients underwent radical resection defined as R0 liver resection [patients who were microscopically positive (R1 liver resection), grossly positive (R2 liver resection), or whose margins were uncertain were excluded]; (d) patients did not die during the perioperative period or were not lost to follow-up after resection; and (e) patients did not receive anti-cancer treatment before surgery. The tumor stage was determined according to the Barcelona Clinical Liver Cancer (BCLC) staging system, while tumor differentiation was determined according to the Edmondson classification system.

### Patient Follow-Up

After collecting peripheral blood samples at admission (7 days before surgery), the patient entered a clinical follow-up period to monitor recurrence and death. Patients underwent various follow-up examinations and treatments according to a routine clinical schedule after surgery. Recurrence was determined based on the results of serum alpha-fetoprotein (AFP) levels, color Doppler ultrasound, computed tomography (CT), magnetic resonance imaging (MRI), digital subtraction angiography (DSA), and positron emission tomography (PET). Recurrence was defined as intrahepatic recurrence and extrahepatic metastases. Recurrence or death was considered as the end point. Follow-up period was from January 1, 2014 to December 31, 2019. The median follow-up time was 52 months. All 214 resectable HCC patients have complete follow-up information.

### CTCs and CTC-WBC Clusters Test

**Figure 2:**
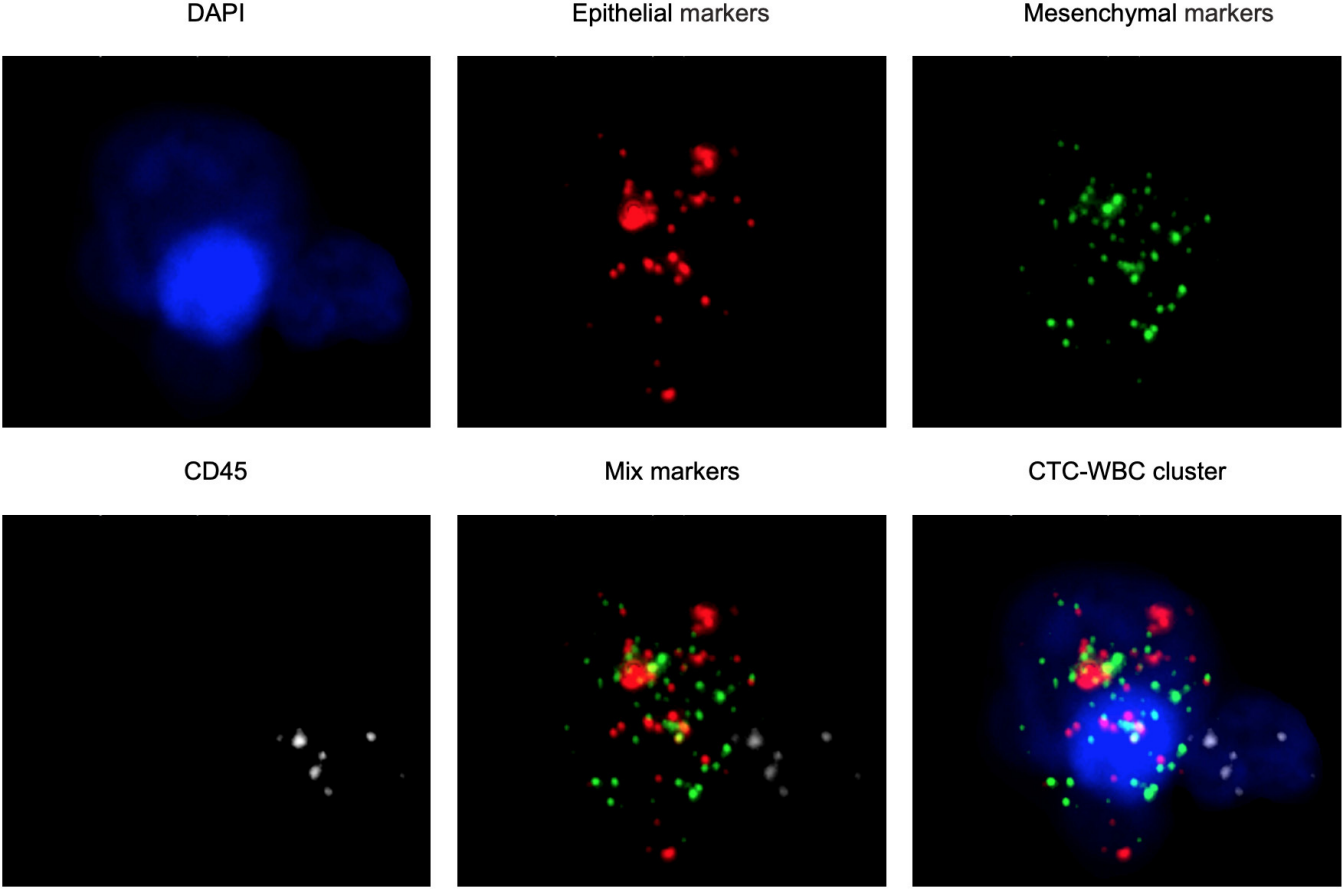
Examples of CTCs, WBCs, and CTC-WBC clusters under automated fluorescent microscope imaging. Epithelial CTCs stained with EpCAM or CK8/18/19 (red). Mesenchymal CTCs stained with Vimentin or Twist (green). WBCs stained with CD45 (white). Nuclei stained with DAPI (blue). DAPI 40,6-diamidino-2-phenylindole.

The CanPatrol™ CTC analysis system (SurExam, China) was used to detect the number of CTCs and CTC-WBC clusters in 7.5 ml of whole samples of peripheral blood, similar to previous studies (17, 18). RNA-ISH was used to detect the following target sequences: white blood cells were labeled with CD45 and visible as white fluorescence, epithelial cells were labeled with EpCAM and CK8/18/19 and visible as red fluorescence, and mesenchymal cells were labeled with vimentin/twist and visible as green fluorescence. Nuclei were labeled with 40,6-diamidino-2-phenylindole (DAPI) and visible as blue fluorescence. CTC-WBC clusters are seen as a white dot of WBCs around a red, green, or red/green mixture of CTCs (Figure 2). After being labeled, the cells were analyzed with a fluorescence microscope.

### Statistical Analysis

PASS version 11 is used to estimate the sample size of survival data 1-year survival rate of patients with CTC-WBC clusters positive and negative hepatocellular carcinoma in our department are estimated to be 72.6 and 84.5%, respectively. The time of all patients enrolled is estimated to be 36 months, and the follow-up time is planned to be 36 months, set α = 0.05 (two-sided), β = 0.2, the ratio between the positive group and negative group is 1:1, and the loss to follow-up rate is 10%. Finally, the estimated total sample size is 142 cases. Categorized data were compared by the Chi-squared or Fisher's exact probability test. The OS and DFS were assessed by Kaplan-Meier analysis using the log-rank test. The Cox proportion hazard regression model was used for the multivariable survival analysis to determine prognostic factors that were significant in univariate analysis for either DFS or OS. All statistical analyses were two-tailed and a *P* < 0.05 was considered to be statistically significant. The data were analyzed using IBM SPSS Statistics 25.0.

## Results

### Clinical and Pathological Characteristics

In the present study, peripheral blood was collected from 214 patients from Zhujiang Hospital for analysis, which is affiliated to Southern Medical University, including 28 women and 186 men, with a median age of 53 (range: 18–78) years. In total, 111 (51.9%) subjects had a tumor with a diameter >5 cm, while the remaining 103 (48.1%) had a tumor with a diameter ≤ 5 cm. While 185 (86.4%) patients had hepatitis B, 110 (51.4%) patients had liver cirrhosis. Preoperative examination showed portal vein tumor thrombosis in 19 (8.9%) patients. Regarding BCLC staging, there were 13 (6.1%) cases of stage 0, 72 (33.6%) cases of stage A, 110 (51.4%) cases of stage B, and 19 (8.9%) cases of stage C. Edmondson staging was performed postoperatively; there were 48 (22.4%) cases of stage I, 67 (31.3%) of stage II, 55 (25.7%) of stage III, and 44 (20.6%) of stage IV. There were 65 (30.4%) cases of encapsulation invasion and 58 (27.1%) cases of microvascular invasion. The cutoff values for total CTCs and CTC-WBC clusters were determined via ROC curve analysis, and the cutoff was considered positive for total CTCs ≥3, CTC-WBC clusters ≥2 (Extended Data Table 1, Figure 1). CTC-WBC clusters and different phenotypic CTC counts showed 141 (65.9%) positive CTCs and 89 (41.6%) positive CTC-WBC clusters. The follow-up period ended on December 31, 2019. There were 156 (72.9%) recurrences and 98 (45.8%) deaths. Table 1 shows the relationship between CTC-WBC clusters and clinicopathological characteristics of HCC. Statistical analysis showed that CTC-WBC clusters were significantly correlated with tumor size (*P* = 0.001), tumor number (*P* = 0.005), portal vein tumor thrombus (*P* = 0.026), BCLC stage (*P* < 0.001), AFP level (*P* = 0.002), and total number of CTCs (*P* < 0.001). However, it was not related to gender, age, liver cirrhosis, Edmondson stage, Tumor encapsulation, Microvascular invasion, and HBsAg.

**Table 1:** Relationship between CTC-WBC cluster and the clinicopathological characteristics of HCC patients.

**Groups**		**CTC-WBC cluster**		**χ^**2**^**	***P***
		**Negative**	**Positive**		
Total	214	125	89		
Gender	Male	111	75	0.938	0.333
	Female	14	14		
Age (years)	<60	89	68	1.262	0.261
	≥60	36	21		
Tumor size (cm)	≤5	72	31	10.795	0.001^*^
	>5	53	58		
Tumor number	Solitary	92	49	7.954	0.005^*^
	Multiple	33	40		
Liver cirrhosis	No	61	43	0.005	0.944
	Yes	64	46		
Portal vein tumor thrombus	No	118	76	4.978	0.026^*^
	Yes	7	13		
BCLC stage	0+A	62	23	12.255	<0.001^*^
	B+C	63	66		
Edmondson stage	I+II	73	42	2.627	0.105
	III+IV	52	47		
Tumor encapsulation	Complete	93	56	3.239	0.072
	None	32	33		
Microvascular invasion	No	97	59	3.365	0.067
	Yes	28	30		
HBsAg	Negative	19	10	0.697	0.404
	Positive	106	79		
AFP (μg/L)	<400	85	42	9.330	0.002^*^
	≥400	40	47		
Total CTCs	Negative	66	7	46.702	<0.001^*^
	Positive	59	82		

* *P < 0.05*.

*BCLC, Barcelona Clinical Liver Cancer; SD, standard deviation; HBsAg, hepatitis B surface antigen; AFP, alpha-fetoprotein; CTCs, circulating tumor cells; CTC-WBC, circulating tumor cell-white blood cells*.

### Survival Analysis

**Figure 3:**
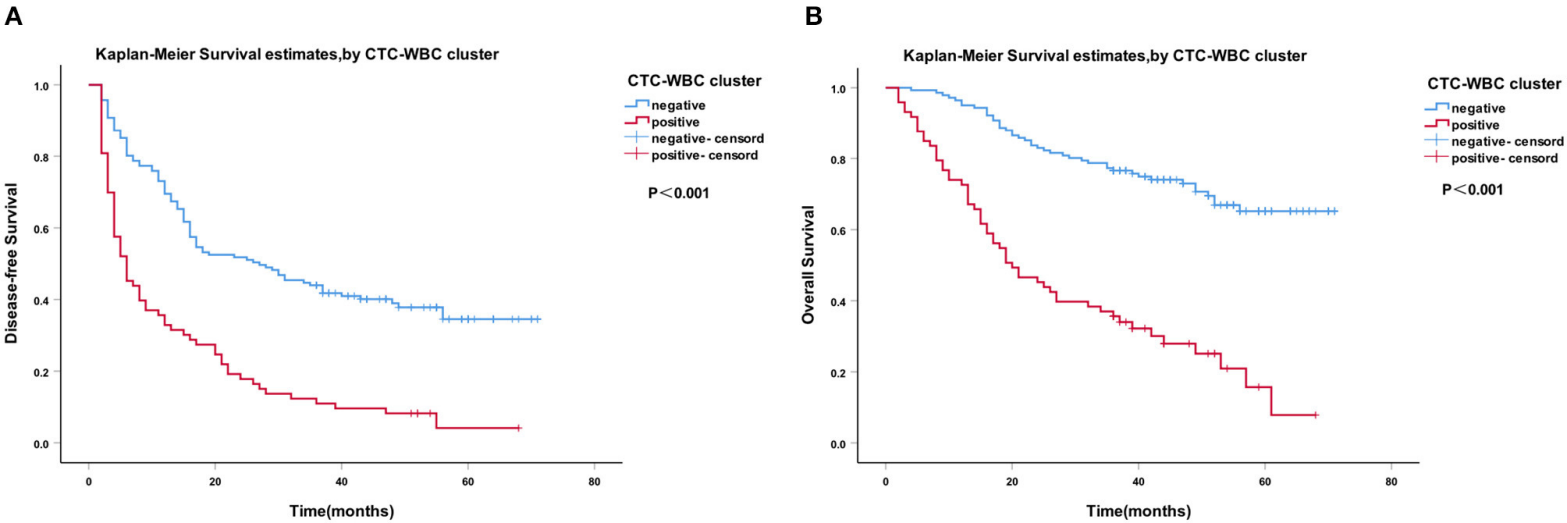
Survival analysis of hepatocellular carcinoma (HCC) patients. CTC-WBC cluster-positive status is associated with a poorer outcome in patients with HCC. Kaplan-Meier analysis revealed significant differences in disease-free survival (*P* < 0.001) **(A)** and overall survival (*P* < 0.001) **(B)** between preoperative CTC-WBC cluster-positive and -negative patients in an HCC cohort. Survival analysis of HCC patients using the Kaplan-Meier method. *P*-values were determined using the log-rank test. DFS, disease-free survival; OS, overall survival. Extended Data Figure 1 ROC curve showing the effectiveness of the use of total CTCs and CTC-WBC clusters in the diagnosis of HCC.

A Cox regression univariate analysis revealed that some factors were associated with the DFS of HCC patients, including CTC-WBC clusters, tumor size, portal vein tumor thrombus, BCLC stage, Edmondson stage, microvascular invasion, AFP level, and total CTCs (Table 2). Some factors were associated with the OS of HCC patients, including CTC-WBC cluster, tumor size, portal vein tumor thrombus, BCLC stage, AFP, and total CTCs (Table 3). A multivariable analysis was performed and the results showed that CTC-WBC clusters (HR = 1.951, 95% CI: 1.348–2.824, *P* < 0.001), tumor size, portal vein tumor thrombus, BCLC stage, AFP and total CTC number were independent predictors of DFS (Table 2). CTC-WBC clusters (HR = 3.026, 95% CI: 1.906–4.802, *P* < 0.001), tumor size, portal vein tumor thrombus, and total CTC number were independent predictors of OS (Table 3). In addition, the Kaplan-Meier curve showed that the DFS (*P* < 0.001) and OS (*P* < 0.001) of HCC patients in the CTC-WBC cluster positive group were shorter than those in the negative group. The 3-year survival rate of the CTC-WBC cluster-positive group was 34.8% and the 5-year survival rate was 17.9%. The 3-year survival rate of the CTC-WBC cluster-negative group was 81.5% and the 5-year survival rate was 70.0% (Figures 3A,B).

**Table 2:** Univariate and multivariable analyses of the predictors of disease-free survival in HCC patients.

	**Univariate analysis**			**Multivariate analysis**		
	**HR**	***P***	**95% CI**	**HR**	***P***	**95% CI**
**Gender**						
Female vs. Male	1.098	0.684	0.699–1.724			
**Age (years)**						
≥60 vs. <60	1.037	0.840	0.728–1.478			
**Tumor size (cm)**						
>5 vs. ≤5	4.785	0.000^*^	3.346–6.841	1.880	0.036^*^	1.041–3.394
**Tumor number**						
Multiple vs. Solitary	1.261	0.163	0.910–1.748			
**Liver cirrhosis**						
Yes vs. No	0.908	0.548	0.664–1.243			
**Portal vein tumor thrombus**						
Yes vs. No	3.998	0.000^*^	2.451–6.522	1.950	0.010^*^	1.176–3.234
**BCLC stage**						
B+C vs. 0+A	4.466	0.000^*^	3.069–6.499	2.078	0.022^*^	1.112–3.882
**Edmondson stage**						
III+IV vs. I+II	1.419	0.029^*^	1.036–1.944	0.926	0.661	0.657–1.305
**Tumor encapsulation**						
None vs. Complete	1.306	0.120	0.932–1.830			
**Microscopic vascular invasion**						
Yes vs. No	1.459	0.033^*^	1.030–2.066	0.832	0.335	0.572–1.209
**HBsAg**						
Yes vs. No	0.851	0.474	0.546–1.325			
**AFP (μg/L)**						
≥400 vs. <400	2.276	0.000^*^	1.655–3.129	1.438	0.045^*^	1.008–2.051
**Total CTCs**						
Positive vs. Negative	3.164	0.000^*^	2.159–4.638	1.675	0.018^*^	1.094–2.563
**CTC-WBC cluster**						
Positive vs. Negative	3.147	0.000^*^	2.276–4.351	1.951	<0.001^*^	1.348–2.824

* *P < 0.05*.

*HR, Hazard ratio; CI, confidence interval; BCLC, Barcelona Clinical Liver Cancer; SD, Standard Deviation; HBsAg, hepatitis B surface antigen; AFP, alpha-fetoprotein; CTCs, circulating tumor cells; CTC-WBC, circulating tumor cell-white blood cells*.

**Table 3:** Univariate and multivariable analyses of the predictors of overall survival in HCC patients.

	**Univariate analysis**			**Multivariate analysis**		
	**HR**	***P***	**95% CI**	**HR**	***P***	**95% CI**
**Gender**						
Female vs. Male	0.615	0.165	0.310–1.221			
**Age (years)**						
≥60 vs. <60	1.447	0.089	0.945–2.216			
**Tumor size (cm)**						
>5 vs. ≤5	7.757	<0.001^*^	4.514–13.331	2.714	0.016^*^	1.202–6.128
**Tumor number**						
Multiple vs. Solitary	1.461	0.065	0.976–2.186			
**Liver cirrhosis**						
Yes vs. No	1.081	0.700	0.727–1.609			
**Portal vein tumor thrombus**						
Yes vs. No	8.167	<0.001^*^	4.722–14.123	3.744	<0.001^*^	2.117–6.622
**BCLC stage**						
B+C vs. 0+A	8.240	<0.001^*^	4.386–15.481	2.374	0.075	0.916–6.153
**Edmondson stage**						
III+IVvs. I+II	1.361	0.127	0.916–2.024			
**Tumor encapsulation**						
None vs. Complete	1.218	0.356	0.801–1.852			
**Microscopic vascular invasion**						
Yes vs. No	1.427	0.102	0.932–2.184			
**HBsAg**						
Yes vs. No	1.002	0.995	0.558–1.798			
**AFP (μg/L)**						
≥400 vs. <400	2.101	<0.001^*^	1.411–3.127	1.039	0.858	0.681–1.586
**Total CTCs**						
Positive vs. Negative	6.697	<0.001^*^	3.559–12.602	2.805	0.003^*^	1.432–5.495
**CTC-WBC cluster**						
Positive vs. Negative	5.347	<0.001^*^	3.471–8.236	3.026	<0.001^*^	1.906–4.802

* *P < 0.05*.

*HR, hazard ratio; CI, confidence interval; BCLC, Barcelona Clinical Liver Cancer; SD, Standard Deviation; HBsAg, hepatitis B surface antigen; AFP, alpha-fetoprotein; CTCs, circulating tumor cells; CTC-WBC, circulating tumor cell-white blood cells*.

## Discussion

We used CanPatrol™ CTC analysis technology to count different types of CTCs and CTC-WBC clusters. CTCs can be divided into different subtypes, including epithelial CTCs, mesenchymal CTCs, and mixed (epithelial/mesenchymal) CTCs. Different subtypes can form CTC-WBC clusters with WBCs. A large number of studies have shown that epithelial-mesenchymal transition (EMT) plays a key role in tumor recurrence and metastasis (19). Our team's previous research also confirmed that mesenchymal CTCs are more ideal early predictors of HCC recurrence (17, 20). However, we found in the study that CTCs in the CTC-WBC clusters were almost all mixed CTCs. This study explored the relationship between CTC-WBC clusters and prognosis in patients with HCC before radical resection. The results showed that CTC-WBC clusters are independent prognostic indicators of DFS and OS in HCC patients. The presence of CTC-WBC clusters in the peripheral blood and CTC-WBC cluster-positive patients have worse prognosis.

CTCs are relatively safe and readily available “liquid biopsy” specimens (21, 22). In fact, CTCs can serve as both an indicator of diagnosis and prognosis and provide molecular information to guide treatment decisions (17, 22, 23). Several studies have shown that CTCs are independent risk factors for HCC, and patients with higher CTC counts have a poorer prognosis (23, 24). The circulating CTCs usually exist in the form of single cells and multiple CTCs can also be clustered together (25). In addition, previous studies have shown that CTC clusters in the peripheral blood have a survival advantage and have enhanced tumor cell metastasis and colonization capacity in both mouse models and patients (25, 26). Do CTCs form clusters with other cells? We first detected CTC-WBC clusters in the peripheral blood of HCC patients using CanPatrol™ CTC analysis technology in 2014. The finding supports the role of tumor-associated immune cells in the development of cancer. It has potential value because the application of immune checkpoint blocking in the treatment of many different types of cancer is ever increasing. However, what role and significance do WBCs in the peripheral blood have for CTCs? It is clear that in a tumor microenvironment, tumor-associated neutrophils (TANs) promote the growth and metastasis of cancer cells through direct effects on cancer cells and indirect effects on tumor cells by changing the tumor microenvironment (27). Zhou et al. found that TANs play a crucial role in tumor development and progression in the tumor microenvironment (28). However, these studies only confirmed that TANs in the primary tumor microenvironment promoted the growth of HCC. It is not clear whether TANs are present in the peripheral blood, and if so, whether it also promotes CTC proliferation. Neutrophils are part of the natural immune system and form the largest proportion of white blood cells (WBC) in the human circulation. In this study, we found that CTC-WBC clusters in the peripheral blood are associated with portal vein tumor thrombi and microvascular invasion. Nonetheless, we do not know whether CTC-WBC clusters are contained in portal vein tumor thrombi and microvessel invasion nests. However, as we know portal vein tumor thrombus and microvascular invasion are independent prognostic factors of hepatocellular carcinoma, this correlation between CTC-WBC cluster and portal vein tumor thrombus or microvascular invasion also suggest that HCC patients with positive CTC-WBC cluster have a poor prognosis. In a recent publication, Szczerba et al. (29) tested for CTCs in blood samples from 70 breast cancer patients. It was found that most CTCs in the circulation were single CTCs and a small number of CTC clusters (8.6%) were CTC-WBC clusters (3.4%). It was confirmed that compared with a single CTCs or CTC cluster, the presence of CTC-neutrophil clusters is related to the poor prognosis of breast cancer patients. We identified the presence of CTC-WBC clusters in the peripheral blood of patients with HCC in 2014 and counted CTC-WBC clusters when classifying patients' peripheral blood CTCs. The 5-year follow-up confirmed that CTC-WBC clusters were related to DFS and OS in HCC patients and were an independent predictor of DFS and OS. In addition, the Kaplan–Meier analysis also showed that CTC-WBC cluster-positive patients lived for a shorter time than the CTC-WBC cluster-negative patients.

## Conclusions

In summary, we found that there are a certain number of CTC-WBC clusters in the peripheral blood of patients with HCC. CTC-WBC clusters are associated with common risk factors such as AFP, total CTC count, portal vein tumor thrombus, and microvascular invasion, and CTC-WBC clusters in the peripheral blood are an independent predictor of DFS and OS and their presence indicate poor prognosis in patients with HCC. This phenomenon gives us a hint that circulating CTCs may have their own immune microenvironment and both the “seed” and “soil” are involved in metastasis. This may open the door to new therapeutic targets directed against cell-cell junctions and associated survival pathways. In addition, we found in the study that CTCs in the CTC-WBC cluster were almost all mixed CTCs; why this was the case is not clear at this time, and is a direction worthy of future research. The results of this study can provide evidence for CTC-WBC cluster as a potential biomarker for the prognosis of HCC. However, The present study had several limitations. First, retrospective cohort study and limited sample sizes have affected statistical power to draw clear conclusions. If this conclusion can be further verified in a follow-up prospective multicenter study, it will be more reliable. Second, it included only chinese patients recruited from a single institution, the achieved results cannot be generalized to other patient populations, especially to non-Asian patients. Finally, we mainly focused on patients with resectable hepatocellular carcinoma after surgery, and data on advanced patients is worthy of further study and discussion.

## Data Availability Statement

The original contributions presented in the study are included in the article/Supplementary Material, further inquiries can be directed to the corresponding author/s.

## Ethics Statement

The studies involving human participants were reviewed and approved by Institutional Review Board of the Second Affiliated Hospital of Southern Medical University (2020-KY-003-01). The patients/participants provided their written informed consent to participate in this study.

## Author Contributions

MP and YC: conception and design. QL and BP: development of methodology. BP, LC, HL, KC, and CZ: acquisition of data. QL and CW: analysis and interpretation of data and writing, review, and/or revision of the manuscript. MP and XP: administrative, technical, or material support. MP and YC: study supervision. All authors contributed to the article and approved the submitted version.

## Conflict of Interest

XP was employed by company SurExam Bio-Tech. The remaining authors declare that the research was conducted in the absence of any commercial or financial relationships that could be construed as a potential conflict of interest.
