# Supplementary material for: Circulating Tumor-Cell-Associated White Blood Cell Clusters in Peripheral Blood Indicate Poor Prognosis in Patients With Hepatocellular Carcinoma
Source: Front Oncol. 2020 Nov 2;10:1758. doi: 10.3389/fonc.2020.01758 (PMC7667255; doi:10.3389/fonc.2020.01758)
Supplement: Supplementary file 1 [file Table_1.DOCX]

Extended Data Table 1. The diagnostic efficiency of total CTCs and CTC-WBC clusters in differentiating patients with HCC

| subtype | Cutoff point | | Sensitivity | Specificity | YI | AUC(95% CI) |
| --- | --- | --- | --- | --- | --- | --- |
| CTC-WBC cluster | | ≥1 | 0.891 | 0.328 | 0.219 | 0.734(0.660–0.807) |
|  | | ≥2 | 0.532 | 0.897 | 0.429 |  |
|  | | ≥3 | 0.404 | 0.914 | 0.318 |  |
| CTC | | ≥2 | 0.981 | 0.207 | 0.188 | 0.713(0.627–0.798) |
|  | | ≥3 | 0.776 | 0.655 | 0.431 |  |
|  | | ≥4 | 0.718 | 0.655 | 0.373 |  |

YI: Youden Index; AUC: Area under the Curve.
