# Supplementary figures and images for: Circulating Tumor-Cell-Associated White Blood Cell Clusters in Peripheral Blood Indicate Poor Prognosis in Patients With Hepatocellular Carcinoma
Source: Front Oncol. 2020 Nov 2;10:1758. doi: 10.3389/fonc.2020.01758 (PMC7667255; doi:10.3389/fonc.2020.01758)

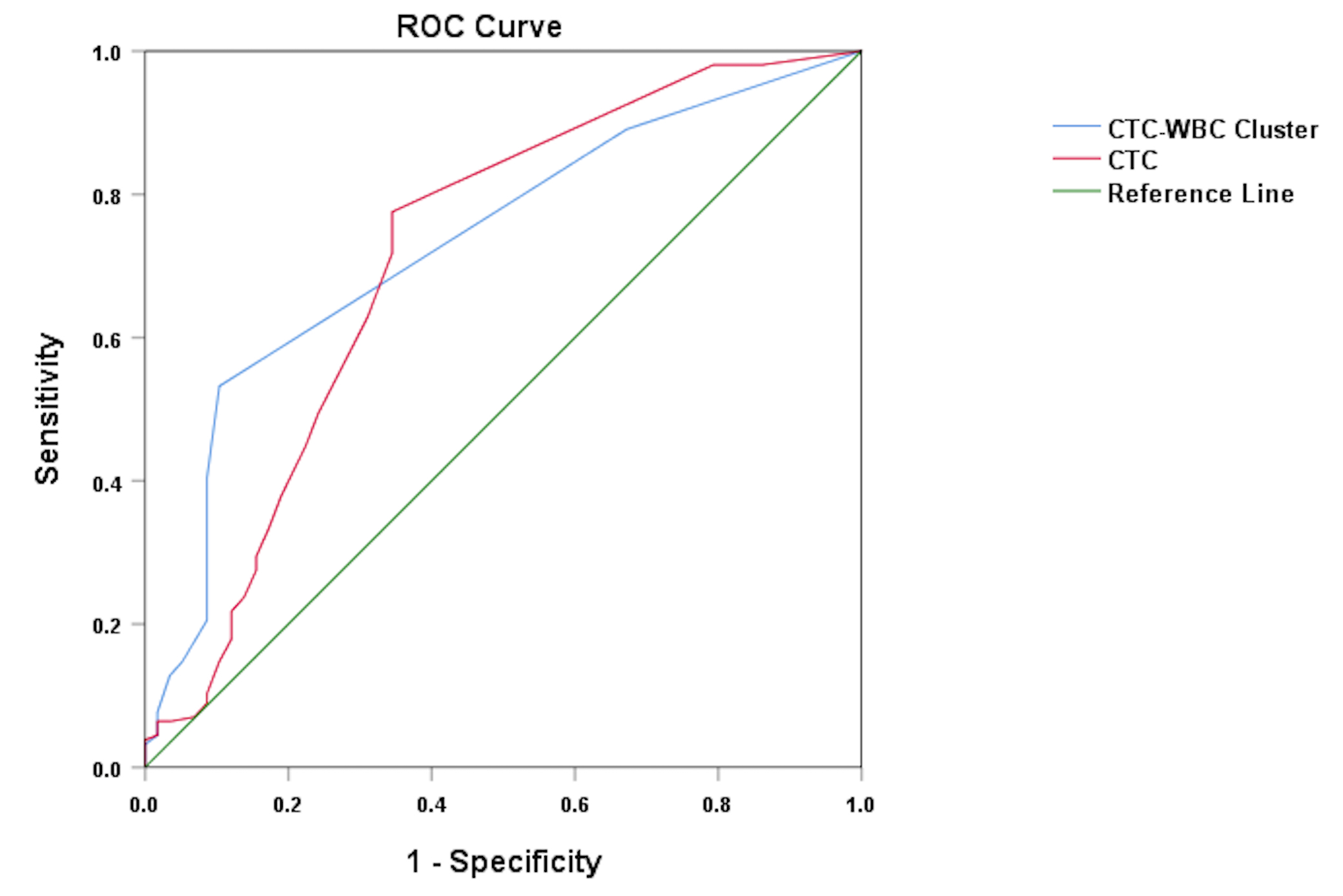

Supplement: Supplementary file 2 [file Image_1.JPEG]
